# Sol-Gel Assisted Immobilization of Alizarin Red S on Polyester Fabrics for Developing Stimuli-Responsive Wearable Sensors

**DOI:** 10.3390/polym14142788

**Published:** 2022-07-08

**Authors:** Valentina Trovato, Alessio Mezzi, Marco Brucale, Hamed Abdeh, Dario Drommi, Giuseppe Rosace, Maria Rosaria Plutino

**Affiliations:** 1 Department of Engineering and Applied Sciences, University of Bergamo, Viale Marconi 5, 24044 Dalmine, Italy; hamed.abdeh@unibg.it (H.A.); giuseppe.rosace@unibg.it (G.R.); 2 Institute for the Study of Nanostructured Materials, ISMN—CNR, Via Salaria Km 29.3, 00015 Monterotondo Stazione, Italy; alessio.mezzi@cnr.it; 3 Institute for the Study of Nanostructured Materials, ISMN—CNR, Via P. Gobetti 101, 40129 Bologna, Italy; marco.brucale@cnr.it; 4 Department of ChiBioFarAm, University of Messina, Viale F. Stagno d’Alcontres 31, Vill. S. Agata, 98166 Messina, Italy; dario.drommi@unime.it; 5 Institute for the Study of Nanostructured Materials, ISMN—CNR, Palermo, c/o Department of ChiBioFarAm, University of Messina, Viale F. Stagno d’Alcontres 31, Vill. S. Agata, 98166 Messina, Italy

**Keywords:** wearable pH-meter, smart textile, sol-gel, alizarin red S, organic–inorganic material, polyester fabric

## Abstract

In the field of stimuli-responsive materials, introducing a pH-sensitive dyestuff onto textile fabrics is a promising approach for the development of wearable sensors. In this paper, the alizarin red S dyestuff bonded with a sol-gel precursor, namely trimethoxy-[3-(oxiran-2-ylmethoxy)propyl]silane, was used to functionalize polyethylene terephthalate fabrics, a semi-crystalline thermoplastic polyester largely used in the healthcare sector mainly due to its advantages, including mechanical strength, biocompatibility and resistance against abrasion and chemicals. The obtained hybrid halochromic silane-based coating on polyester fabrics was investigated with several chemical characterization techniques. Fourier transform infrared spectroscopy and X-ray Photoelectron Spectroscopy confirmed the immobilization of the dyestuff-based silane matrix onto polyethylene terephthalate samples through self-condensation of hydrolyzed silanols under the curing process. The reversibility and repeatability of pH-sensing properties of treated polyester fabrics in the pH range 2.0–8.0 were confirmed with diffuse reflectance and CIELAB color space characterizations. Polyester fabric functionalized with halochromic silane-based coating shows the durability of halochromic properties conversely to fabric treated with plain alizarin red S, thus highlighting the potentiality of the sol-gel approach in developing durable halochromic coating on synthetic substrates. The developed wearable pH-meter device could find applications as a non-invasive pH sensor for wellness and healthcare fields.

## 1. Introduction

Smart textiles are wearable materials, able to convert stimuli into responses, interacting dynamically with the environment. In recent years, they have emerged as attractive resources due to the tremendous potential background in the Internet of Things (IoT) and military, space, healthcare or wellness applications [1,2,3,4,5,6]. As a result, widespread research and development have been carried out, making them an indispensable part of an emerging new technology field. Among others, pH-sensitive wearable devices are currently attracting interest in several areas of fundamental and applied research with the goal of detecting acidic or alkaline values for real-time monitoring applications in healthcare [7,8,9]. Indeed, wearable chemical sensors may be used to provide valuable information about wearers’ health, monitoring them during their daily routine.

Among available platforms for developing “smart” sensors, clothing is of valuable interest since it is form-fitting for direct skin contact and worn by everyone.

Besides several properties making fabrics interesting for realizing intelligent platforms, such as low cost, flexibility and other unique characteristics like wettability, breathability and mechanical and chemical resistance, specific thermal requirements can be binding for choosing the most suitable fabric according to the application.

For example, fibres for medical applications must be non-toxic, non-allergic, non-carcinogenic and sterilizable without suffering chemical or physical damage. While cotton is widely used for applications requiring hydrophilicity, it has been replaced by synthetic fibres for several technical applications due to more advantageous characteristics, such as durability, low linting features and inert characteristics [10].

Polyester (PL), especially polyethylene terephthalate, is the most widely employed of all synthetic fibres due to its high strength, excellent physical and mechanical properties, abrasion and wrinkle resistance, stiffness, tailorable performance and relatively low cost [11]. Moreover, the use of polyester fibres is widely diffuse for medical applications as a consequence of their high modulus, high strength, low creep and biocompatibility, for example, as both non-implantable and implantable materials (e.g., artificial tendon, artificial ligament, vascular grafts, artificial kidney, aortofemoral grafts and many extra-anatomic bypass grafts) [12].

Examples of pH sensing coatings were developed on polyester fabrics using branched polymer nanoparticles by dip-coating [13], or [poly(acrylic acid)] and [poly(2-vinyl pyridine)] by an epoxide-containing polymer [14], or plasma-assisted sol-gel treatment [15]. Furthermore, halochromic dyestuffs were used in electro-spinning and impregnation processes to investigate the sensing behavior of different treated textile materials [16]. Unfortunately, despite many beneficial properties, polyester shows several disadvantages, like poor dyeability and adhesion of finishing associated with its non-polar and hydrophobic nature, resulting in poor washing fastness of applied coatings [17]. Thus, it is essential to design polyethylene terephthalate fabrics with excellent coating adhesion performance in order to enhance the durability. In several studies, polyester was modified either by chemical hydrolysis or by irradiation to introduce functional groups onto the surface, which increases the hydrophilicity of the polymer before the application of chemicals [18,19]. However, to avoid such pre-treatments, in the present work, PL samples were treated with a sol-gel-based silane precursor as a crosslinker able to promote the formation of a complex hybrid network exhibiting adhesion forces with the substrate. Indeed, after hydrolysis, siloxane groups are involved in condensation reactions with themselves and with hydroxyl and/or carboxyl groups at the polyethylene terephthalate polymer ends. These groups on the polyester surface can facilitate the formation of chemical bonds between the fibres and hybrid segments with a siloxane backbone linked to a halochromic dyestuff. This versatile approach permits the combining of organic functional components with hydrolyzed metal precursors, leading to stable colloidal solutions. The latter is used to develop a hybrid xerogel coating suitable for polyester finishing due to its tunable thickness and porosity, optical transparency, proton permeability and biocompatibility.

Among alkoxysilane sol-gel precursors, trimethoxy-[3-(oxiran-2-ylmethoxy)propyl]silane (GPTMS) is widely used for developing hybrid silane-based textile finishing [20]. The presence of both inorganic and organic units covalently linked to each other makes GPTMS of relevant interest since it can promote the formation of extended cross-links between the alkoxysilane network and the silanol groups, thus enhancing adhesion upon coated materials through the epoxy-ring opening.

To the best of our knowledge, few studies on silane-functionalized alizarin red S covalently immobilized onto textile fabrics are reported in the literature [21]. These studies are exclusively related to the functionalization of cotton and do not consider the interaction between sol-gel precursors and fibres characterized by low hydrophilic nature, high degree of crystallinity and a poor number of chemically reactive groups.

With this goal, in this research, polyester fabrics realized through ARS-based silane functionalization were fully characterized by several physical–chemical techniques to investigate the adhesion and durability of applied coating, as well as its halochromic behavior. The success of grafting reactions and changes in the surface chemical composition of fabrics were studied by Fourier transform infrared spectroscopy (FTIR) and X-ray Photoelectron Spectroscopy (XPS). Changes in the morphology of treated fabrics were characterized with optical, scanning electron and atomic force microscopies. In addition, diffuse reflectance spectroscopy and CIELAB color space measurements were used to ascertain the pH-sensing proprieties of developed polyester fabrics, as well as their reversibility and repeatability after several laundering cycles and pH exposures. As a matter of fact, the functionalization of polyester fabrics with halochromic dyestuff through GPTMS can provide useful wearable pH sensors for potential applications in advanced materials and medical fields thanks to the biocompatibility of polyester textiles and non-cytotoxicity of the alkoxysilane matrix [22].

## 2. Materials and Methods

### 2.1. Materials, Preparation of Halochromic Fabrics and Method of Testing

Polyethylene terephthalate, in the following coded as polyester fabric (PL, mass per unit area of 213 g/m^2^, Cittadini S.p.A, Paderno Franciacorta, Italy), was selected as textile substrates for developing stimuli-responsive wearable sensors. With the aim of removing impurities before treating fabrics, textiles were washed with a non-ionic detergent for 20 min (40 °C, pH 7), then thoroughly rinsed with deionized water and dried in a convection oven.

Pure alizarin red S (ARS) and silane-modified alizarin red S (GPTMS-ARS), prepared according to the procedure described in our previous research [21], were used to treat textile fabrics (21 cm × 29 cm) by simple pad dry-cure method using a two-roll laboratory padder (Werner Mathis, Zurich, Switzerland) by setting up a nip pressure of 3 bar. After impregnation, the wet treated textile fabrics were subjected to subsequent drying (90 °C, 5 min) and curing (170 °C, 3 min) steps. The obtained textiles were coded as PL_ARS and PL_GPTMS-ARS, respectively.

The halochromic behavior of treated fabrics was assessed using buffer solution (2 < pH < 8) prepared using phosphoric acid (85%), disodium hydrogen phosphate dodecahydrate (Na_2_HPO_4_ • 12 H_2_O) and sodium dihydrogen phosphate dihydrate (NaH_2_PO_4_ • 2 H_2_O) (purchased from Carlo Erba).

For each treated fabric, the total dry solid add-on (wt%) was calculated according to Equation (1):

(1) $$Add-on\;\left(\%\right)=\frac{W_{T}-W_{UT}}{W_{UT}}\times 100$$

where *W_T_* and *W_UT_* are the dry weights of treated and untreated fabrics, respectively.

The washing fastness of treated samples and the leaching of both pure ARS and silane-modified ARS dyestuffs from fabrics were investigated by calculating the weight loss of fabrics after washing cycles (*WLW*, wt%) according to Equation (2):

(2) $$WLW\;\left(\%\right)=\frac{W_{TW}-W_{T}}{W_{T}}\;\times \;100$$

where *W_T_* and *W_TW_* are the dry weights of treated and treated-washed fabrics, respectively. With this aim, treated textile samples were weighted after 1 and 5 washing cycles (sample codes “1W” and “5W”, respectively) performed in Labomat Mathis equipment (Werner Mathis AG, Oberhasli, Switzerland) according to the ISO 105C01, and *W_TW_* and *W_T_* are the dry weights of treated fabrics after 1 or 5 launderings and before washing, respectively. All treated and untreated samples were conditioned under 65 ± 4% relative humidity at 20 ± 2 °C, for at least 24 h in a climatic chamber, before all the experiments. For calculating both add-on (%) and WLW (%), all textile fabrics were weighted three times using a Mettler balance (accuracy 10^−4^ g) with a standard deviation lower than 2%.

### 2.2. Chemical–Physical Characterizations

To study the chemical–physical properties and morphology of the treated polyethylene terephthalate fibre surface, as well as the efficacy and reliability of the developed halochromic coatings, fabric samples were investigated with several characterization techniques.

Attenuated total reflectance (ATR) Fourier transform infrared (FTIR), thanks to its penetration depth of a few micrometres, allows studying the surface chemical composition of materials, and therefore it was used to obtain information on the functional groups characterizing the surface of coatings applied on textile fabrics, as well as their adhesive properties. A Thermo Avatar 370 equipped with a diamond crystal as an internal reflectance element was used to collect spectra of textile fabrics in the range 4000–550 cm^−1^ (with a resolution of 4 cm^−1^ and after 32 scans). To remove the shifting of the entire spectrum, the intensity of the FTIR-ATR spectra was normalized using the IR band at 1339 cm^−1^ [23,24], assigned to the stretching vibration in the carboxylic group of polyethylene terephthalate fibres, which remains unaffected during surface modification.

Moreover, the surface chemical composition of both untreated and treated fabrics was investigated by X-ray Photoelectron Spectroscopy (XPS), using an ESCALAB MkII (VG Scientific, East Grinstead, UK) spectrometer, equipped with a standard Al excitation source and 5-channeltron as detection system [25]. The experiments were carried out with the constant pass energy set to 50 eV and the binding energy (BE) scale was calibrated, positioning the C 1s peak from adventitious carbon at BE = 285.0 eV. All spectra were registered and processed with Thermo Avatange software v5.979 (Thermo Fisher Scientific Ltd., East Grinstead, UK).

The morphology of all samples at the millimetre, micrometre and nanometre scale was studied with Optical Microscopy (OM), Scanning Electron Microscopy (SEM) and Atomic Force Microscopy (AFM), respectively.

Images with Optical Microscopy were taken using a Nikon Eclipse 80i microscope (Nikon, Tokyo, Japan) with a Plan Fluor objective operated in a reflected brightfield. SEM images were acquired on a Hitachi S4000 SEM operated at 15 kV, mounting textile samples onto an aluminium stub (PELCO) and coating them with a ≈ 10 nm thick 99.999% Au (Sigma-Aldrich) layer by means of a sputter coater (Quorum QR150R). A Bruker Multimode8 microscope was used for AFM characterization of fabrics. The instrument is equipped with a Nanoscope V controller and a JV-type piezoelectric scanner. SNL-A probes (Bruker Probes, Camarillo, CA, USA) were used to collect images in PeakForce mode and the background subtraction was performed in Gwyddion 2.58 [26].

UV-Vis diffuse reflectance was performed on washed textile samples (to avoid non-bonded dyestuff interference) after soaking in pH buffer solution and drying to assess the optical absorption properties and the pH response of polyethylene terephthalate fabrics treated with plain and silane-functionalized ARS. For determining the pH of textile samples, a single-extraction operation was carried out from textiles impregnated for 5 min in buffer solutions at different pH values, using 250 mL of bidistilled water and 10 g of textile sample. The pH measurement of the extracted solution was carried out potentiometrically. Since the pH value thus obtained was the same as the used buffer solution, it was taken as the pH of the textile material.

The stimuli-responsive behavior of treated fabrics was investigated with UV-Vis diffuse reflectance spectroscopy after 1 and 5 washing cycles to avoid non-bonded dyestuff interference.

To study the halochromic properties of treated textiles, each sample was soaked in pH buffer solutions in the pH range between 2 and 8. A double beam UV-Vis scanning spectrophotometer (Thermo Nicolet Evolution UV–Vis 500, Waltham, MA, USA), equipped with a diffuse reflectance accessory (RSA-PE150 Labsphere), was used to acquire reflectance spectra of textile samples (50 mm × 70 mm) in the spectral region between 380 nm and 770 nm with 10 nm interval.

Each sample was analyzed three times, and collected reflectance measurements (experimental error of approximately 2%) were averaged (R, %) and used in Equation (3) to calculate the Kubelka–Munk values:

(3) $$\frac{K\;\left(\lambda \right)}{S\left(\lambda \right)}=\frac{\left(1-R_{\infty }\right)}{2R_{\infty }}$$

In Equation (3), the absolute reflectance of an effectively infinitely thick layer is *R_∞_*, whereas the adsorption and scattering coefficients are *K* and *S*, respectively. Both adsorption and scattering coefficients were also involved in the calculation of the dye fixation ratio (*F_dye_*, %) of developed fabrics following Equation (4), which is an index of color strength.

(4) $$F_{dye}\left(\%\right)=\frac{{\left(\frac{K}{S}\right)}_{TW}}{{\left(\frac{K}{S}\right)}_{T}}\times 100$$

where *(K/S)_T_* and *(K/S)_TW_* are color-strength values at the maximum absorbance wavelength of treated samples before and after washing cycles, respectively.

To study the halochromic properties of developed fabrics, the washed fabrics were analyzed with CIE (International Commission of Illumination) L*a*b* color-space measurements using a standard illuminant D65/10° for the acquisition of color-space coordinates, L* (lightness), a* (position between green and red) and b* (position between yellow and blue). In accordance with these values, a textile sample can be assigned to a defined color region. Accordingly, green and red colors are due to negative and positive a* values, whereas blue and yellow colors are due to negative and positive b* values [27].

The total color difference between two samples is defined by ΔE* (Equation (5)):

(5) $$\Delta E^{*}=\sqrt{{\left(\Delta L^{*}\right)}^{2}+{\left(\Delta a^{*}\right)}^{2}+{\left(\Delta b^{*}\right)}^{2}}$$

In this equation, ΔL*, Δa* and Δb* are the brightness, redness and yellowness differences between two samples [27,28]. According to the literature [28,29], when ΔE* is greater than 1 or 12, the color difference between the two samples is perceptible by the naked eye or belongs to a different space, respectively.

CIE L*a*b* calculations were also performed on treated polyester samples to investigate the repeatability of the silane-based ARS sensors in comparison with those of plain ARS.

For this experiment, five repeated pH-exposure cycles (pH 2 and 8) of textiles were performed. Each cycle consists of the soaking of the textiles in pH 2 buffer solution (5 min). Thus, samples were dried and soaked again in pH 8 solution (5 min) and finally dried. For each fabric, and after each pH-exposure cycle, the differences of the color space coordinates between pH 2 and 8 for all samples were measured by ΔE*.

## 3. Results and Discussion

Developed halochromic polyester fabrics were investigated with different physical–chemical characterizations to investigate the feasibility and advantages of the sol-gel approach in designing durable and reliable wearable sensors.

### 3.1. ATR-FTIR Characterization and Washing Fastness Evaluation of Dyed Textile Fabrics

**Figure 1:**
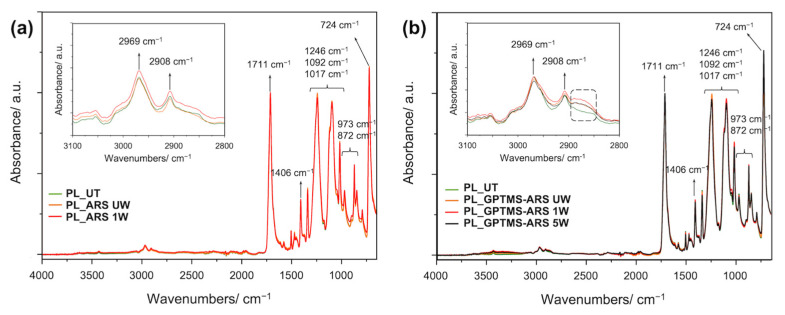
ATR-FTIR spectra of pristine polyester fabrics (PL_UT), dyed with plain ARS (**a**) and treated with silane-functionalized ARS (**b**) before (_UW) and after 1 and 5 washing cycles (_1W and _5W, respectively).

Infrared spectroscopy was used to investigate the chemical composition of the developed halochromic polyester fabrics. Textile samples treated with plain ARS and silane-functionalized ARS were characterized before and after washing cycles (1 and 5 wash) to assess the durability of the halochromic coatings. In Figure 1, FTIR spectra of unwashed (coded as “UW”) and washed (coded as “1W” and “5W”) treated fabrics are reported in comparison with untreated polyester samples (coded as “UT”).

From an ATR-FTIR spectra comparison between treated and untreated polyester fabrics (Figure 1a,b), new absorption bands assigned to the coatings are not readily visible since they overlap with the intense infrared peaks of polyester. Indeed, the main characteristic peaks of polyester fabrics highlighted in Figure 1a,b, assigned to C=O stretching in ester (1711 cm^−1^), C=C stretching in benzene (1406 cm^−1^), C–O stretching in ester and carboxylic acid (1246 cm^−1^, 1092 cm^−1^, 1017 cm^−1^), in/out of plane CH=CH bending and C–H bending (973 cm^−1^, 872 cm^−1^ and 724 cm^−1^) [24,30,31], cover absorption bands of silane network. However, the new broadband at around 2850 cm^−1^, assigned to the C–H stretching mode ascribable to the GPTMS-alkyl chain, confirms the surface modification of polyester fabrics by trimethoxy-[3-(oxiran-2-ylmethoxy)propyl]silane (Figure 1b, inset).

To better assess the advantage of the sol-gel approach in the development of durable wearable sensors, Equation (1) was used to calculate the add-on (wt%), which describes the silane coating adhesion onto textile substrates with respect to plain-ARS-treated fabrics. Moreover, following Equations (2) and (4), the durability of the dyeing (both with ARS and silane-functionalized ARS) in terms of weight loss, and the fixation ratio (F_dye_, %) were studied, respectively.

The higher add-on (wt%) value reported in Table 1 highlights that the silane functionalization of ARS provides stronger adhesion of the dyestuff on polyester substrates compared to that observed for plain ARS. Moreover, the silane functionalization of ARS provides certain durability of the coating with respect to the unfunctionalized ARS. Indeed, non-durability of the plain ARS coating was suggested by the WLW calculation and images of PL-ARS fabrics since samples showed complete dye leaching and the loss of color properties even after the first washing cycle due to the weak linkage between textile surfaces and plain dyestuff. For this reason, in Table 1 and for every proposed characterization, data referred to polyester ARS-dyed fabrics after five laundry cycles are not reported.

**Scheme 1:**
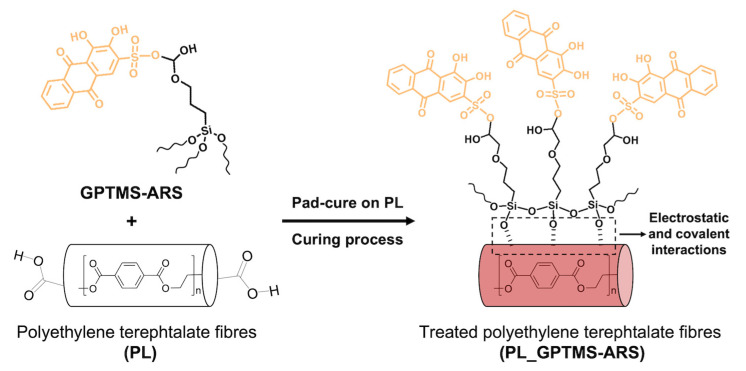
Schematic representation of GPTMS-ARS pad-cure process on PL fibres.

Compared to natural fibres like cotton, silk and wool and to some manmade ones like polyamide and rayon showing good wettability due to functional chemical groups such as –NH_2_, –COOH and –OH in their polymer chains, polyester exhibits low hydrophilicity and low wettability. These characteristics are due to the synthetic procedure of polyester involving polycondensation of terephthalic acid and ethylene glycol, thus resulting in fibres with high crystallinity degree with a poor number of chemically reactive groups. However, the existence of hydroxyl and/or carboxyl groups at the polymer ends can promote the formation of chemical bonds and electrostatic interactions between the ARS-containing alkoxysilane precursors and fibres. Once coatings are applied by padding, the solution is instantly absorbed by inter-fibre spaces. Thereupon, a variety of intermolecular forces such as H-bond, van der Waals forces and dipole–dipole interactions [32], resulting from the ARS-GPTMS structure molecules and polarized bonds on polyethylene terephthalate chains, increase the adhesion properties of applied finish (Scheme 1).

Following these observations, good results can be expected from treated samples by modifying their surfaces with the presence of trimethoxy-[3-(oxiran-2-ylmethoxy)propyl]silane to increase the adhesion of ARS to the fabric surface, promoting the formation of a highly firm network on the polyester fabric surfaces compared to the plain alizarin red S. Accordingly, ARS-containing silane coating shows a durability 98% higher than pure ARS, with a result (WLW = 2.33%) similar to that calculated in another study [21] for highly reactive fibres, such as cotton (WLW = 1.05%).

**Scheme 2:**
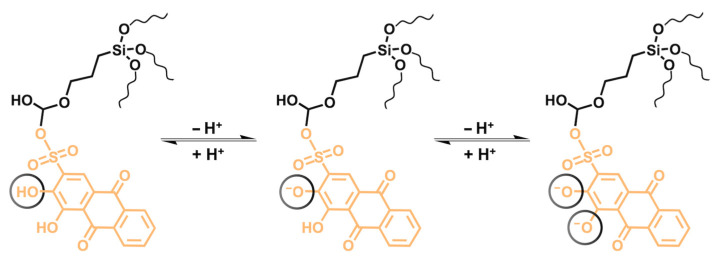
Alizarin red S resonance structure at different pH values corresponding to neutral, mono-anionic and di-anionic forms.

Supporting the gravimetric measurements performed on treated polyester samples to investigate the coating durability, reflectance spectroscopic characterizations were performed for calculating the coating color strength through the fixation ratio F_dye_ (%) (using R values at the maximum absorbance wavelength). As already observed in previous research studies [21,33,34], the alizarin red S molecule exists in different resonance structures according to the pH media due to its pK_a_ values at 5.49 and 10.85. In acidic media (pH < 5), ARS is in the neutral form, characterized by both hydroxyl groups being protonated, while when increasing pH media, the mono-anionic and dianionic forms are observable for pH ranging between 5 and 9 and greater than 9, respectively (Scheme 2). In particular, for the investigated pH range in this study (between 2 and 8, where GPTMS-ARS-treated samples show their absorption maxima), the two characteristic maxima absorbance wavelengths at around 423 nm and 520 nm, corresponding to the neutral and mono-anionic forms, respectively, were observed.

Although relatively low wettability was expected for polyester fabrics towards halochromic silane-based coating, the fixation ratio calculated for GPTMS-ARS-treated polyester fabrics showed a loss of color strength between 1 and 5 washing of approximately 29.9%. This loss is comparable to that shown by more reactive substrates, such as cotton, treated with the same silane-based coating (ΔF_dye_ = 28.4%) [21], confirming a certain affinity of such silane-based coating toward polyester fabrics.

### 3.2. Surface Chemical Composition of Textile Fabrics by XPS

**Figure 2:**
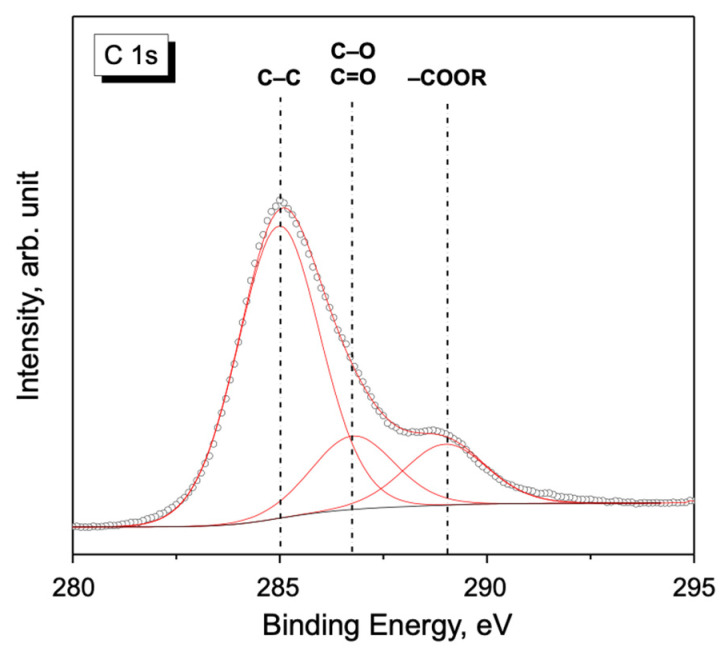
C 1s spectra of PL_UW samples.

The surface chemical composition of textile fabrics was analyzed by XPS before and after coating deposition and after washing cycles. Table 2 shows XPS results obtained for polyester fabrics. For all polyester samples (treated with both plain ARS and GPTMS-ARS), the presence of C and O was assessed, while for those treated with silane-functionalized ARS, the presence of Si was also investigated. In Figure 2 the C 1s spectra of PL_UT samples fitted by adding three synthetic peaks positioned at BE = 285.0 eV, 286.6 and 288.9 eV (assigned to C–C, C–O, C=O and COOR bonds, respectively [35]) are reported.

For the O 1s signal, a single broad peak that includes the whole surface oxygen species –OH, C=O and Si–O was observed. The effectiveness of the silane-functionalized ARS coating was confirmed by the presence of the Si on treated samples, as suggested by the BE value of the Si 2p peak positioned at BE = 102.7 eV. This value suggests that the C–Si–O bond in the RSiO_3_ configuration is responsible for the adhesion of the silane coating on polyester surfaces.

The presence of the silane coating is revealed by the detection of a Si 2p peak on the surface of treated samples compared with the untreated PL surface (Table 2). Even after washing cycles, XPS results still showed the presence of the silane coating on polyester samples, also highlighted by the Si/C ratio that is not influenced by laundering, thus confirming the coating durability. Furthermore, the Si/C ratio of ARS-GPTMS-treated polyester samples confirms the poor uptake of silane-functionalized halochromic dyestuff in agreement with the low wettability of polyester fibres [21].

### 3.3. Morphological Characterization of Untreated and Treated Textiles

**Figure 3:**
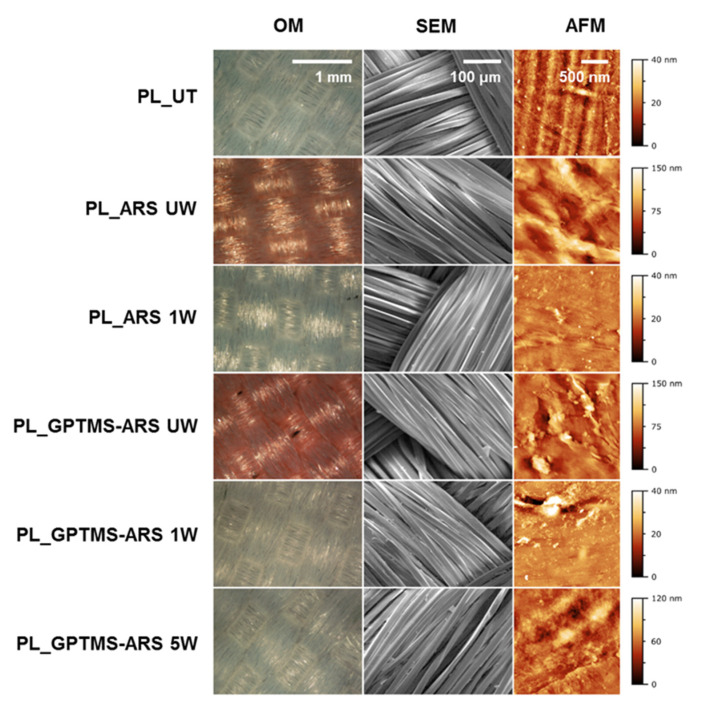
Images of developed halochromic polyester fabrics with Optical Microscopy (OM), Scanning Electron Microscopy (SEM) and Atomic Force Microscopy (AFM).

Surface characterizations with OM, SEM and AFM were carried out on both plain-ARS- and silane-functionalized-ARS-treated polyester fabrics to investigate morphological variations at millimetre, micrometre and nanometre scales (Figure 3).

OM images reported in Figure 3 evidence that all PL samples are characterized by fibres woven in an orderly manner with a low degree of protrusion or breakage and no structural differences among polyester samples regardless of dyeing treatments (if plain ARS or GPTMS-ARS) or washing cycles.

Investigation of micrometre-scale morphology via SEM imaging evidenced PL samples uniformly showing an ordered and uniform arrangement of smooth fibres. However, no discernible structural modification was apparently induced by any functionalization and washing steps at this length scale.

In contrast, AFM revealed that both coatings led to nanoscale morphology changes. PL fibres showed a slightly corrugated but continuous surface by exposing a surface area of 1.02 ± 0.02 µm^2^ per projected square micrometer. PL samples dramatically changed their roughness following functionalization with both ARS and GPTMS-ARS. While individual untreated PL fibrils have an average RMS roughness (S_q_) of 4.2 ± 0.5 nm, unwashed PL_ARS and PL_GPTMS-ARS fibres have an S_q_ = 23 ± 6 nm and 25 ± 7 nm, respectively. This suggests that the deposition of coating material upon functionalization occurred in random aggregates, of which only a fraction is attached to the underlying substrate. As a further confirmation, washing cycles have a pronounced effect on PL samples, resulting in severe material loss after just one washing. Accordingly, S_q_ values of PL_1W samples returned to values similar to those of the untreated fabric, although a qualitative inspection of AFM micrographs suggested that a small fraction of the material still adheres to the substrate.

### 3.4. Diffuse Reflectance Measurements: Textile pH Response

In the field of wearable sensors, properties such as sensor performance, reversibility and repeatability are fundamental for the development of a robust and reliable sensor capable of providing a dynamic response to a specific analyte (e.g., pH).

With this aim, unwashed and washed ARS and GPTMS-ARS-treated polyester fabrics were investigated through UV-Vis diffuse reflectance spectroscopy to assess the durability of their pH-response properties. K/S equivalent absorption units of polyester samples were calculated according to equation 3 by selecting maxima absorption peaks observed for each sample after the washing cycles (Table 3) and fixing the same wavelength for both unwashed and washed samples. Unwashed textiles and washed fabrics treated with plain ARS reveal maxima absorbance peaks at a lower wavelength than those observed for washed samples and GPTMS-ARS. These findings are according to the covalent functionalization of the silane-modified ARS that influences the chemical structure of conformers since the modification in the chromophore electron delocalization leads to changes in the halochromic behavior [36].

**Figure 4:**
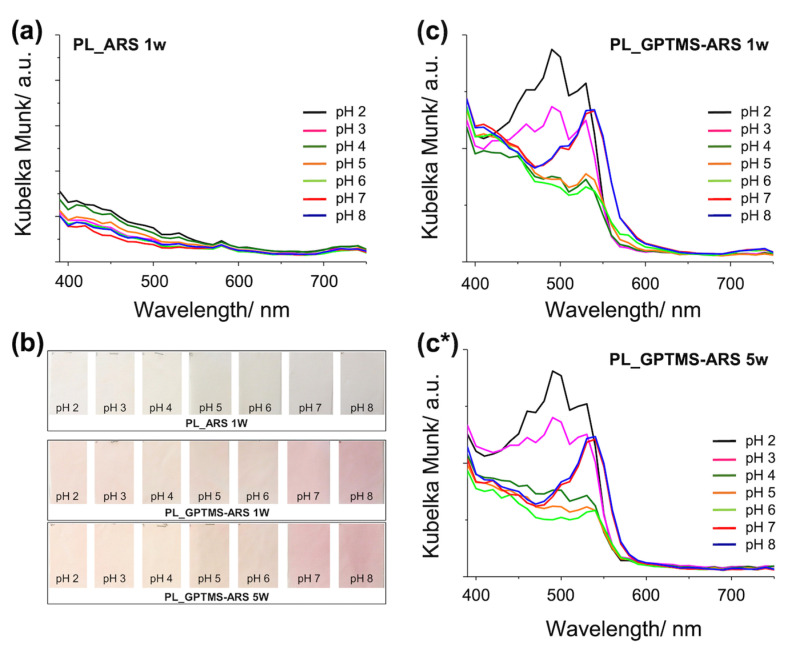
Kubelka–Munk spectra of polyester fabric treated with plain ARS after 1 wash (**a**), with GPTMS-ARS after 1 and 5 washing cycles (**c** and c*, respectively) at different pH values. Images of PL_ARS and PL_GPTMS-ARS after 1 and 5 washing cycles (**b**).

Data reported in Table 3 evidenced the percentage loss in the halochromic properties for polyester fabrics treated with plain ARS after washing cycles. In particular, almost total dye leaching was observed for polyester fabrics after washings (PL_ARS 1W and 5W) since no maxima should be discerned in the corresponding spectra. Conversely, GPTMS-ARS-treated polyester samples showed a loss in the halochromic properties equal to 94.4% and 96.3% after 1 and 5 washing cycles, respectively, as a result of the breaking of bonds between the ARS-containing coating and the textile surface during washing cycles. Nevertheless, these fabrics revealed a significant pH response, as highlighted by images and K/S curves reported in Figure 4.

The diffuse reflectance spectroscopic characterization of polyester fabrics treated with plain ARS (Figure 4a) evidenced almost no pH response after one washing cycle, which is confirmed by the digital images of treated fabric soaked in buffer solutions with pH ranging from 2 to 8 (Figure 4b). Therefore, spectra and images of PL_ARS 5w are not reported since the dye release was otherwise too high to allow for a halochromic study. Unfortunately, Kubelka–Munk curves of polyester samples treated with silane-functionalized ARS (Figure 4c,c*) present an irregular trend, probably due to the low concentration of GPTMS-ARS coating present on the textile surface after washing cycles. Nevertheless, the Kubelka–Munk curves of the GPTMS-ARS-treated polyester fabrics differ according to the pH, highlighting a maximum absorbance wavelength at around 500 nm for pH 2 and 3, moving to 530 nm for pH 7 and 8.

### 3.5. Dynamic Response of Halochromic Coatings to Changes in pH

**Figure 5:**
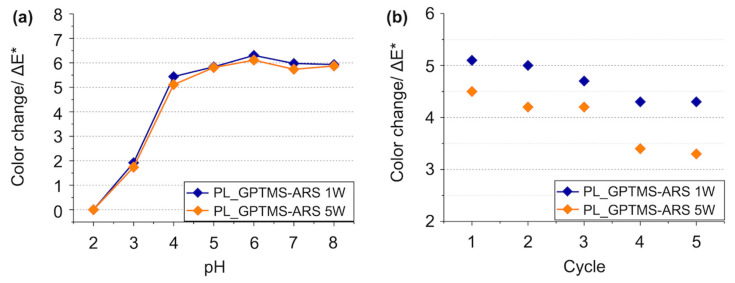
ΔE* color change with respect to pH 2 of PL_ARS, PL_GPTMS-ARS after 1 and 5 washing cycles (blue and orange curves, respectively) as a function of pH variations (**a**) and repeated cycles (**b**).

Both dynamic response of treated polyester fabrics and the consistency of their halochromic properties were studied by recording changes in CIELAB color space (ΔE*) in the pH range between 2 and 8, where GPTMS-ARS-treated samples show their absorption maxima, thus neglecting any color changes related to other pH changes, and data are reported in Figure 5.

Since ARS-dyed polyester fabrics did not show any pH response, these samples were not tested for dynamic response to pH changes. Conversely, the pH-sensing properties of polyester fabrics were observed when coated with the silane-functionalized ARS even after several washing cycles.

To study the dynamic response of treated polyester fabrics, textiles were alternatively soaked into buffer solutions with pH values between 2 and 8, defining the response time within 5 min and showing a distinct color variation in this pH range. In Figure 5a, the recorded color changes in CIELAB color space (ΔE*) for each sample, normalized as a function of ΔE* at pH 2, are reported. Since ΔE* is the perceptual color difference between two measurements in CIE L*a*b* color space, for higher measured ΔE* values, more evident halochromic changes can be observed. Experimental findings reported in Figure 5a suggest a good color fastness of PL-GPTMS-ARS samples since the measured ΔE* is still constant after one and five washing cycles, thus confirming the durability of the silane-based coating.

Treated fabrics were also exposed to repeated cyclic exposure to acid/alkaline buffer solutions to investigate the consistency of their halochromic properties. Accordingly, textiles were submerged firstly in acid buffer solution (pH 2) and then in alkaline buffer (pH 8), after washing with bidistilled water, and the difference between color space values of these samples was calculated. Fabrics were exposed to this cyclic exposure to acid/alkaline solution five repeated times and ΔE* for each cycle is reported in Figure 5b.

As evident by the difference in color space calculated for each pH value reported in Figure 5, the silane-functionalized ARS coating present on treated polyester fabrics is not significantly affected by the number of washing cycles.

The low wettability and the high crystalline structure, as well as the low number of polar groups on polyethylene terephthalate textiles, seem not to affect the covalent functionalization of the silane-based ARS significantly, thus resulting in a certain dynamic pH response even after the first washing cycle, as confirmed by the ΔE* curves reported in Figure 5b.

## 4. Conclusions

In this research study, GPTMS alkoxysilane-functionalized alizarin red S was used as a halochromic sensing molecule to coat polyester fabrics. As a result, the synthetic fabrics treated with the optically transparent organic–inorganic halochromic film showed pH-responsive color change. Indeed, the developed silane-treated polyester fabrics showed a certain dynamic response to pH changes even after washing cycles compared to plain-ARS-treated ones that completely leach the halochromic molecules after the first washing cycle. Moreover, investigations on coating adhesion performed by gravimetric and spectrophotometric calculations confirm the formation of cross-links between the alkoxysilane network and the surface functional groups of polyester fabrics.

Experimental findings suggest that sol-gel can be efficiently used as a coupling agent for stimuli-responsive dyestuff to improve coating adhesion onto polyester fabrics, reaching high desired properties and good washing fastness of the finishing compared to plain dyestuff. Results highlight the great potential of the designed synthesis for the immobilization of ARS onto polyethylene terephthalate fabrics, for developing wearable halochromic sensors.

Furthermore, the ecological approach to developing wearable sensors sensitive to external stimuli could increase the use of polyester as a technical fabric for healthcare, sport and medical applications.

## Figures and Tables

**Table 1:** Images and add-on (wt%), weight loss (WLW, wt%) and fixation ratio (F_dye_, %) data of textile fabrics treated with plain ARS solution and GPTMS-ARS sol calculated at the maxima absorbance wavelength as reported in the footnote (^1^).

	PL_ARSZ		PL_GPTMS-ARS	
**Add-on (%)**	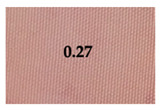		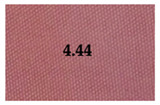	
	1W	5W	1W	5W
**WLW (%)**	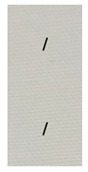	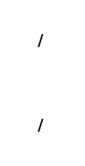	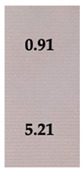	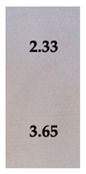
**F_dye_ (%)**				

^1^ PL_ARS λ_max_ = N.A. (no clearly defined maxima peaks); PL_GPTMS-ARS λ_max_ = 530 nm.

**Table 2:** Surface chemical composition (at %) of untreated polyester samples (PL_UT) and polyester samples treated with ARS (PL_ARS) and hybrid silane ARS (PL_GPTMS-ARS) before and after washing cycles (UW, 1W and 5W, respectively) with XPS analysis (10 % standard deviation).

	Sample Code						
Chemical Composition (at %)	PL_UT	PL_ARS			PL_GPTMS-ARS		
		UW	1W	5W	UW	1W	5W
**C–C**	52.0	45.5	42.6	-	43.9	41.4	41.9
**C–O, C=O**	13.1	13.8	14.3	-	15.3	16.7	16.4
**COOR**	10.9	11.3	10.8	-	9.6	10.7	10.8
**SiO_2_, O–Si–C**	-	-	-	-	2.8	2.9	2.8
**Si/C**	-	-	-	-	0.04	0.04	0.04

BE (eV): 285.0 (C–C), 286.8 (C–O, C=O), 288.9 (COOR), 102.7 (SiO_2_, O–Si–C).

**Table 3:** K/S of the treated polyester fabrics at the peak maximum as reported in the footnote (^1^).

Sample	Unwashed Sample	After 1 Washing Cycle	After 5 Washing Cycles
PL_ARS	1.07	-	-
PL_GPTMS-ARS	1.08	0.06	0.04

^1^ PL_ARS λ_max_ = 500 nm (only for unwashed samples); PL_GPTMS-ARS λ_max_ = 530 nm.

## Data Availability

The data presented in this study are available in the article.
